# Myocardial Infarction and Azygos Vein Thrombosis After ChAdOx1 nCoV-19 Vaccination in a Hemodialysis Patient

**DOI:** 10.7759/cureus.18390

**Published:** 2021-09-30

**Authors:** Chun-Yen Chiang, Chin-Yu Chen, Wen-Liang Yu, Wei-Chih Kan, Yin-Hsun Feng

**Affiliations:** 1 Department of Cardiology, Chi Mei Medical Center, Tainan, TWN; 2 Department of Optometry, Chung Hwa University of Medical Technology, Tainan, TWN; 3 Department of Radiology, Chi Mei Medical Center, Tainan, TWN; 4 Department of Intensive Care Medicine, Chi Mei Medical Center, Tainan, TWN; 5 Department of Nephrology, Chi Mei Medical Center, Tainan, TWN; 6 Department of Medical Laboratory Science and Biotechnology, Chung Hwa University of Medical Technology, Tainan, TWN; 7 Department of Hematology and Oncology, Chi Mei Medical Center, Tainan, TWN

**Keywords:** vaccine-induced immune thrombotic thrombocytopenia, chadox1 ncov19 vaccine, hemodialysis, azygos vein thrombosis, myocardial infarction

## Abstract

Vaccine-induced immune thrombotic thrombocytopenia (VITT) is a rare complication after vaccination of Oxford-AstraZeneca coronavirus disease 2019 (COVID-19) vaccine (AZD1222) or Janssen COVID-19 vaccine. It makes a rare complication of thrombosis at common and/or uncommon organs with thrombocytopenia after COVID-19 vaccination four to 28 days later and most patients were younger than 60 years of age. We reported the case of a 75-year-old female with end-stage renal disease who received regular hemodialysis. She received Oxford-AstraZeneca COVID-19 vaccination eight days ago and then she suffered from intermittent chest tightness and epigastric pain with tarry stool passage for two days. Severe thrombocytopenia with elevated D-dimer value was noted and computed tomography of the chest showed azygos vein thrombosis. Elevated cardiac enzyme with ST-T change in 12-lead electrocardiogram was also noted. For positive anti-platelet factor 4 antibodies, VITT with myocardial infarction and azygos vein thrombosis was diagnosed.

## Introduction

The outbreak of severe acute respiratory syndrome coronavirus 2 (SARS-CoV-2) impacts all people in the world since January 2020. It spreads faster and gets severe diseases followed by coronavirus 2019 (COVID-19) variants changing. Fast-vaccination of COVID-19 is ongoing in most countries in the pandemic era of COVID-19 since this year. However, cases were reported as the novel syndrome of vaccine-induced immune thrombotic thrombocytopenia (VITT) after vaccination with the recombinant adenoviral vector encoding the spike protein antigen of SARS-CoV-2[1-3]. From a UK cohort study, most patients with VITT were younger than 60 years of age and the mortality was higher in patients with thrombocytopenia and intracranial hemorrhage [4]. We reported the case of a 75-year-old female who has coronary artery disease and chronic renal failure with hemodialysis received Oxford-AstraZeneca COVID-19 vaccination eight days ago before first symptoms. She suffered from chest tightness and epigastric discomfort with gastrointestinal bleeding and visited the emergency room. Elevated cardiac enzyme and ST-T change on the 12-lead electrocardiogram were noted and myocardial infarction (MI) was diagnosed. The patient has new onset of severe thrombocytopenia with a positive anti-platelet factor 4 autoantibody, and the computed tomography of the chest showed uncommon azygos vein thrombosis. VITT-related MI and azygos vein thrombosis were diagnosed.

## Case presentation

A 75-year-old female is a patient of chronic renal failure in end-stage renal disease with regular hemodialysis since March 2021. She has a history of type 2 diabetes mellitus and coronary artery disease with our cardiovascular outpatient department follow-up. This time she developed chest tightness and epigastric pain accompanied by tarry stool for two days, and she visited the emergency department of our hospital.

**Figure 1:**
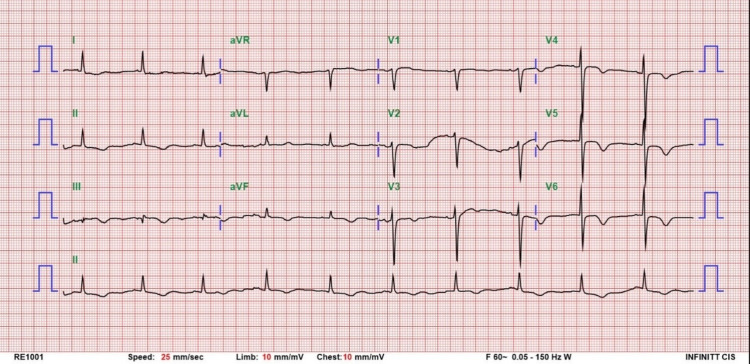
Twelve-lead electrocardiogram at emergency department aVR: augmented vector right; aVL: augmented vector left; aVF: augmented vector foot

At the emergency department, the vital signs of the patient were blood pressure of 133/88 mmHg, heart rate of 78 beats per minute, and body temperature of 36.2℃. The troponin I was elevated with 5518 pg/ml and 12-lead electrocardiogram (ECG) showed new onset of T wave inversion over leads II, III, and augmented vector foot (aVF) and V3-V6 compared with the previous 12-lead ECG of the patient (Figure 1). Thrombocytopenia (platelet count: 57,000/ul) and anemia (hemoglobin: 7.1 g/dl) were noted. The prothrombin time level was 10 (international normalized ratio {INR}: 0.91) and the activated partial thromboplastin time peak was 0.7. Blood transfusion with two units of packed red blood cells (PRBC) was done for anemia. Troponin I was up to 34,300 pg/ml and non-ST elevation acute myocardial infarction (MI) was diagnosed, and she was admitted to the coronary care unit (CCU) for intensive care. The result of the real-time reverse transcription-polymerase chain reaction of SARS-CoV2 of the patient was negative. The physical examination was unremarkable except for abdominal tenderness. She received upper gastrointestinal (GI) panendoscopy for upper GI bleeding which showed peptic ulcer disease with bleeding. We used ticagrelor only and stopped Bokey and heparinization later because of active GI bleeding and thrombocytopenia of the patient.

**Figure 2:**
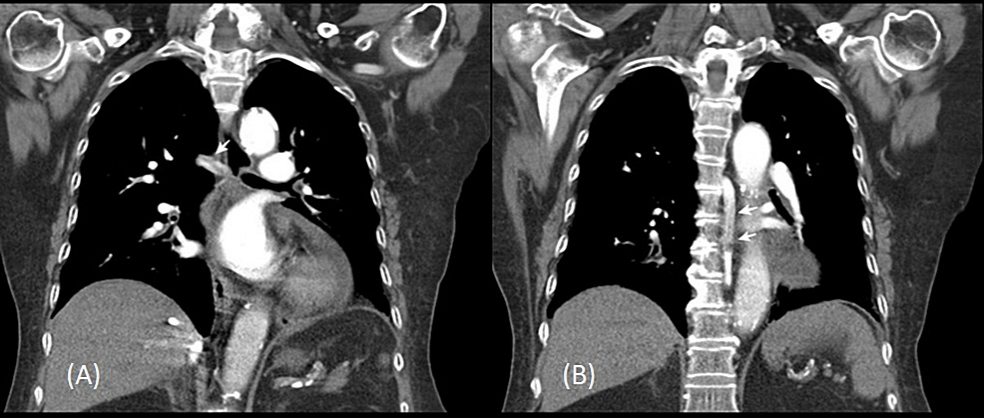
The post-contrast enhanced chest CT in coronal reformation showed partial filling defect in the horizontal (A) and vertical (B) portions of the azygos vein, indicating azygos vein thrombosis (white arrows)

During the CCU stay, a series of examinations were done (Table 1). The patient received blood transfusion of PRBC and platelet on the first day of CCU for active and persistent GI bleeding. Aspartate aminotransferase was 217 unit/l and alanine aminotransferase was 331 unit/l at CCU day three. The D-dimer was up to 5102 ng/ml at CCU day five. Persistent thrombocytopenia with a platelet count of 41,000/mm^3^ was also noted at CCU day three. The abdominal ultrasound showed no liver cirrhosis and there was no hepatitis B and no hepatitis C detected. The chest computed tomography (CT) and abdomen CT were done to evaluate pulmonary embolism and splanchnic vein thrombosis. The chest CT image showed partial azygos vein thrombosis (Figure 2). We reviewed her medical records and there was no thrombocytopenia or previous thrombosis or preexisting prothrombotic condition before and no other etiology-related thrombocytopenia was identified. The autoantibody of platelet factor 4 was checked by enzyme-linked immunosorbent assay on the second day of CCU and the result showed 54.41 pg/ml and optical density value was 0.526 (normal range <0.4). Throughout the hospitalization course, she had severe thrombocytopenia after the first dose of COVID-19 vaccination of ChAdOx1 nCoV-19 (AstraZeneca) eight days ago. Vaccine-induced thrombotic thrombocytopenia (VITT)-related to MI and azygos vein thrombosis was diagnosed according to the serological results and image finding of the patient. At CCU, cardiac catheterization was declined by the patient. Then she was transferred to the cardiovascular (CV) ward on the sixth day of hospitalization.

**Table 1:** Timeline of clinical and laboratory characteristics of the patient ER: emergency room; OPD: outpatient department; PRBC: packed red blood cells; PH: platelets pheresis; cryo: cryoprecipitate; CK: creatine kinase; CKMB: creatine kinase isoenzyme MB mass

Timeline	June 4, 2021	June 16, 2021	June 24, 2021	June 25, 2021	June 26, 2021	June 27, 2021	June 28, 2021	June 29, 2021	July 1, 2021	July 2, 2021	July 5, 2021	July 7, 2021	July 9, 2021	July 10, 2021	July 24, 2021
		Vaccination day	ER/Admission day 1 (ICU)	Day 2 (ICU)	Day 3 (ICU)	Day 4 (ICU)	Day 5 (ICU)	Day 6 (ward)	Day 8 (ward)	Day 9 (ward)	Day 12 (ward)	Day 14 (ward)	Day 16 (ward)	Discharge	OPD
Blood transfusion			PRBC 4 units	PRBC 2 units	PH 1 unit					Cryo 12 unit	PRBC 2 unit				
Platelet count (per mm^3^)	193,000		57,000	60,000	41,000	60,000	56,000	67,000		122,000	213,000	201,000	192,000		237,000
Aspartate aminotransferase (U/l)	140				217	93	75	68	36				37		25
Alanine aminotransferase (U/l)	169		148		331	242	202		116				32		20
D-dimer (mg/l)				1400			5102		5188		5168	5092	5079		2407
Fibrinogen (mg/dl)				206.7			372.5								
Hemoglobin (g/dl)	11.8		7.1	8.6	10.9		9.2								
Leukocyte (per mm^3^)	4200		12,600		6700		4100								
Prothrombin time (seconds)			10.2										15.1		
Activated partial thromboplastin time (seconds)			23.1	30	34.9								29		
International normalized ratio			0.7	0.89											
CK (ng/ml)			185	132											
CKMB (U/l)			21.9	14.4											
Troponin I (pg/ml)	228.2		34380.7	34380.7											
C-reactive protein (mg/dl)	4.7			23.6			27.1						10		

At the CV ward, heparin-free hemodialysis was done for the patient. In addition, she suffered from diarrhea and gross hematuria for several days and ever received blood transfusion with 12 units of cryoprecipitate and two units of PRBC without any sequelae. The platelet count raised to within normal range after a series of follow-up of platelet count. We tried low-dose warfarin therapy with a target prothrombin-time ratio of 1.2 to 1.5 for her azygos vein thrombosis after stabilization of GI bleeding and hematuria and platelet count recovery because the patient was unable to receive a direct oral anticoagulant for her chronic renal failure under hemodialysis. In addition, we kept ticagrelor only for her MI for the higher bleeding risk of the patient. After treatment, there was no more abdomen pain or chest pain at the ward. Her GI bleeding and hematuria also improved. She was discharged after 16 days of hospitalization and received regular outpatient department (OPD) follow-up. Any event or rehospitalization was not observed during two months follow-up at OPD.

## Discussion

Over five million people in Taiwan have received the first dose of the Oxford-AstraZeneca COVID-19 vaccine since March 2021 [5]. However, some people suffered from adverse effects after the COVID-19 vaccination [6]. We reported the case of a 75-year-old female on hemodialysis who suffered from VITT with azygos vein thrombosis and MI. Because the patient received hemodialysis and had severe GI bleeding, it was difficult to give the patient standard therapy by non-heparin-based anticoagulant. The use of low-dose warfarin might be an alternative option without strong evidence to treat azygos vein thrombosis following platelet count recovery in the patient. To our knowledge, the patient is the first case of hemodialysis to be presented with VITT-related azygos vein thrombosis and MI. Conservative treatment was done for her azygos vein thrombosis without intravenous immune globulin (IVIG). In addition, medication therapy was done only for her MI. The patient has MI one year ago and she received heparinization at that time, and she also received heparin bolus during hemodialysis three months ago. Then no thrombocytopenia of the patient was noted after heparin exposure. During the hospitalization of the patient, she received blood transfusion of platelet at ICU first day for her active GI bleeding and thrombocytopenia. The patient received further studies of thrombocytopenia including VITT during hospitalization. So we suggest VITT should be considered when patients have thrombocytopenia with common and/or uncommon thrombosis after the COVID-19 vaccination of Oxford-AstraZeneca COVID-19 vaccine (AZD1222) or Janssen.

Azygos vein connected superior and inferior vena cava, and it also provides collateral to blood flow from venous blood to the heart. Azygos vein thrombus is a rare phenomenon and contrast CT is a gold standard method to diagnose azygos vein thrombosis. In general, patients with azygos vein thrombosis have minor symptoms. Anticoagulants are used to treat azygos vein thrombosis, and further investigation for azygos vein thrombosis is recommended.

The incidence of ChAdOx1 nCoV-19-related VITT has also been reported around 0.5-6.8 cases per 100,000 vaccinees [7]. The mortality of VITT cases from series reports was around 30-60% [1-4]. However, most reported VITT patients are younger females aged below 60 years [4]. The current suggestion of VITT treatment by World Health Organization (WHO) guidance is a non-heparin anticoagulant and high dose IVIG [7]. In this case, the patient is an older female under regular hemodialysis and had just minor symptoms of azygos vein thrombosis. Although there was no suggestion of related guidance or study in patients of hemodialysis with VITT of azygos vein thrombosis and MI, we tried low-dose warfarin and ticagrelor after the GI bleeding stabilization and platelet count recovery, and non-heparin-based anticoagulant was not used in the patient. The study's limitation is that the treatment given to the patient in this case has never been studied and thus is not recommended. 

## Conclusions

We reported the case of a 75-year-old female who has chronic renal failure under hemodialysis and was noted severe thrombocytopenia and higher D-dimer after ChAdOx1 nCoV-19 vaccination eight days later. Further image study and serological studies were done to evaluate VITT when clinical suspicion. However, the treatment of VITT of azygos vein thrombosis and MI in the case needed multidisciplinary evaluation of the patient's condition with VITT-related comorbidity.
